# Bile Cast Nephropathy Due to Hepatitis A-induced Hyperbilirubinemia: A Case Report and Literature Review

**DOI:** 10.7759/cureus.35779

**Published:** 2023-03-05

**Authors:** Khalid Ahmed, Fouad Jaber, Lakshmi Pappoppula, Elmkdad Mohammed, Mark M Aloysius

**Affiliations:** 1 Internal Medicine, The Wright Center for Graduate Medical Education, Scranton, USA; 2 Internal Medicine, University of Missouri Kansas City, Kansas City, USA

**Keywords:** acute kidney injuryt, viral hepatitis a, cholemic nephrosis, bile cast nephropathy and renal failure, bile cast nephropathy

## Abstract

Bile cast nephropathy (BCN) or cholemic nephrosis (CN) is a form of acute renal dysfunction that occurs in the setting of hepatic dysfunction and hyperbilirubinemia. We present a case of a 58-year-old woman with a four-day history of intractable nausea, vomiting, and yellowish discoloration of her skin and eyes. Laboratory workup was notable for elevated total bilirubin (mainly direct), liver enzymes, creatinine, and blood urea nitrogen (BUN). The ultrasonography (US) of the abdomen showed hepatic steatosis. The hepatitis panel was remarkable for hepatitis A IgM. She was initially treated with supportive therapy. However, her bilirubin levels reached over 20 mg/dl, creatine was >8 mg/dl, and her estimated glomerular filtration rate (eGFR) was <10. Kidney biopsy showed pigmented casts consistent with BCN. She was started on hemodialysis with significant improvement in her symptoms and liver enzymes. This case underscores the importance of a broad differential diagnosis in cases with hyperbilirubinemia and acute kidney injury. BCN requires renal biopsy for a definitive diagnosis, and these patients usually require hemodialysis.

## Introduction

Cholestasis secondary to liver disease can lead to kidney injury. The mechanism of renal dysfunction in cholestatic liver disease may be attributed to several factors. These include hemodynamic changes such as intravascular depletion, hepatorenal syndrome, or acute tubular necrosis (ATN) [1]. Elevated bilirubin levels can cause direct cytotoxicity to renal tubular cells. In severe cases, the bile acids can precipitate into casts and cause ATN, which may result in renal failure requiring renal replacement therapy [2,3]. This condition is known as bile cast nephropathy (BCN) or cholemic nephrosis (CN). This entity of kidney disease has not yet received much attention in the medical field. Bile casts on renal biopsy in a patient with hyperbilirubinemia and elevated creatinine are pathognomonic for BCN or CN [4]. The tubular injury in this disease can be reversible, so timely detection is critical to determining prognosis in these patients [5]. We report a case of BCN in a patient who developed acute kidney injury secondary to hyperbilirubinemia due to hepatitis A.

The case was presented as a poster presentation at the American College of Gastroenterology (ACG) in Charlotte, North Carolina in 10/2022.

## Case presentation

A 58-year-old woman presented with a four-day history of intractable nausea, vomiting, and yellowish discoloration of her skin and eyes. She denied abdominal pain, fever, chills, hematemesis, hematochezia, diarrhea, or constipation. Her medical history included type 2 diabetes mellitus, hypothyroidism, hypertension, and hyperlipidemia. The patient had traveled to Arizona two months earlier but denied any sick contacts, insect bites, herbal supplements, use of antibiotics, drugs, alcohol/tobacco, or tattooing.

The patient was hemodynamically stable on presentation. Physical exam was notable for scleral icterus. The abdomen was not tender without rebound tenderness or rigidity. Laboratory values were remarkable for elevated total bilirubin, mainly direct, elevated liver enzymes, and high INR (Table 1). In addition, creatinine and blood urea nitrogen (BUN) were elevated (Table 1). Urine analysis was remarkable for elevated urobilinogen.

**Table 1:** Laboratory values *Remarkable laboratory values on admission, on discharge, and three months after discharge ALP: alkaline phosphatase; ALT: alanine transaminase; AST: aspartate aminotransferase; N/A: not available

Labs	Results	Follow-up labs on discharge	Follow-up labs 3 months after discharge	Reference range
Total bilirubin (mg/dl)	7.9	20.5	1.4	0.1-1.2
Direct bilirubin (mg/dl)	6.1	N/A	N/A	<0.3
ALT (U/L)	4792	76	17	4-36
AST (U/L)	5228	63	23	8-33
ALP (U/L)	313	189	120	44-147
Creatinine (mg/dl)	2.8	8	0.8	0.59-1.04
Blood urea nitrogen (mg/dl)	32	78	12	10-20
Estimated glomerular filtration rate (mL/min/1.73m^2^)*	19	6	89	90-120
International normalized ratio (INR)	2.35	0.97	N/A	0.8-1.1

**Figure 1:**
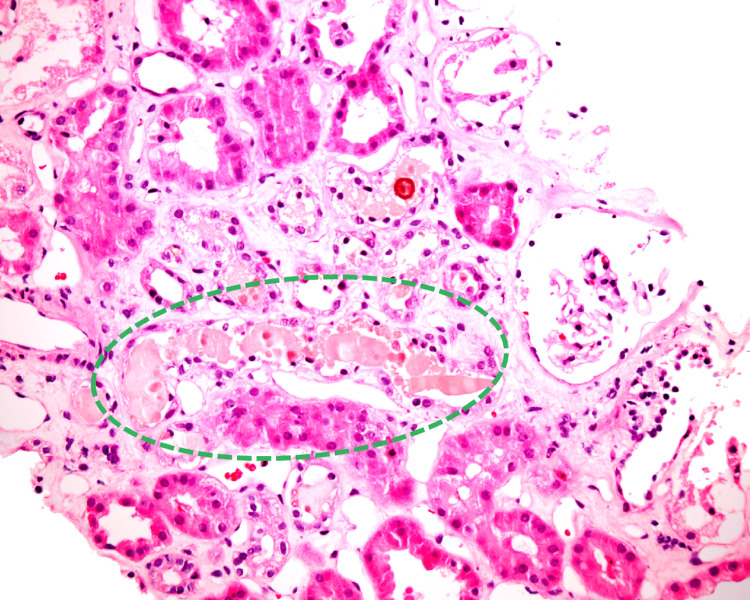
Kidney biopsy (hematoxylin and eosin ×400) A representative area of kidney biopsy showing acute tubular necrosis and bile pigments in tubular epithelial cells (encircled)

**Figure 2:**
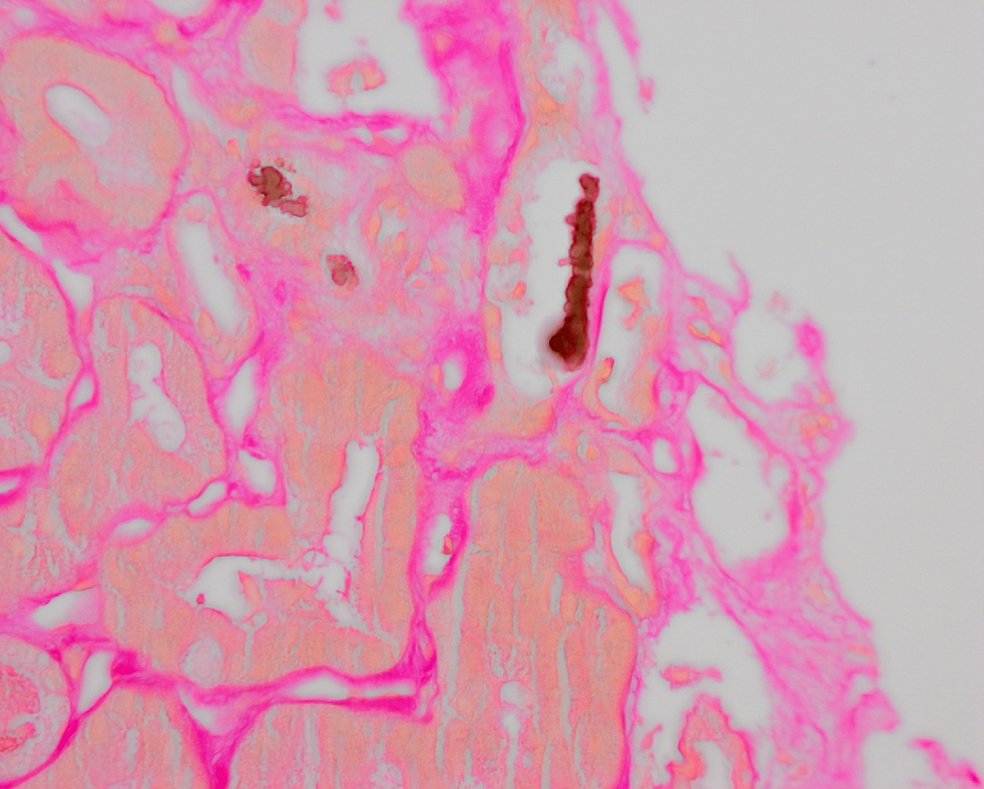
Kidney biopsy (Fouchet stain ×400) A representative area of kidney biopsy with Fouchet stain showing positivity of bile pigments

CT scan of the abdomen and pelvis without contrast revealed evidence of hepatic steatosis. IgM against hepatitis A was positive, while IgM anti-HEV, HBsAg, and anti-HCV were negative. The management included supportive therapy. In the following days, bilirubin levels reached over 20 mg/dl, creatine was >8 mg/dl, and her estimated glomerular filtration rate (eGFR) was <10. The patient was initiated on hemodialysis with significant improvement in her symptoms. Percutaneous ultrasonography (US)-guided left kidney core biopsy showed acute tubular injury with frequent pigmented casts, consistent with BCN or CN (Figure 1). A Fouchet stain for bile was positive within focal intralobular casts (Figure 2).

On the day of discharge, liver enzymes improved, but bilirubin remained elevated (Table 1). Outpatient hemodialysis was scheduled, a follow-up appointment was arranged, and the patient was discharged home. Repeat laboratories three months later showed normalization of liver enzymes, total bilirubin, and kidney functions (Table 1).

## Discussion

BCN is a form of acute renal dysfunction in the setting of liver dysfunction and hyperbilirubinemia [3]. Jaundice-related nephropathy can lead to renal failure, referred to as CN [2]. The pathophysiology of BCN remains unclear, but several hypotheses exist. In humans, cholesterol is a precursor to the production of bile acids in the liver [6]. These bile acids go through enterohepatic circulation, are excreted in the bile, released into the intestines, and then recycled back to the liver [7]. These bile acids can also leak into the systemic circulation, where they are delivered to the kidneys and reabsorbed into the circulation by the proximal convolute tubules (PCT) and delivered to the liver. Once cholestasis develops, liver cells upregulate pumps that help eliminate excess bile. In addition, a similar process takes place in the PCT of the kidneys to excrete bile [4]. This excess of bilirubin is thought to cause oxidative damage to kidney tubular cells by disrupting mitochondrial phosphorylation. This process can lead to tubular hypertrophy and hyperplasia, as suggested by Holmes in 1953, who performed autopsies on 68 patients with hyperbilirubinemia, with 73.5% having these findings [8]. In addition, tubular obstruction can also occur due to bile cast deposition. This process is enhanced by the ability of bile acids to promote cast formation by altering pH via inhibition of Na+/H+, Na+/K+, and Na+/Cl transporter [4].

A literature review of BCN secondary to hepatitis A-induced hyperbilirubinemia revealed four cases (Table 2). All cases involved male patients, and their ages ranged from eight to 35 years; however, in our case, the patient was a 58-year-old female. The most common presenting manifestations were fever and jaundice. Laboratories showed elevated ALT, AST, bilirubin, and creatinine.

**Table 2:** A literature review of BCN cases due to hepatitis A-induced hyperbilirubinemia ALP: alkaline phosphatase; ALT: alanine transaminase; AST: aspartate aminotransferase; ATN: acute tubular necrosis; BCN: bile cast nephropathy; HAV: hepatitis A virus; HCV: hepatitis C virus; HEV; hepatitis E virus; N/A: not available

Variables	Khatri et al., 2022 [9]	Khiangte et al., 2019 [10]	Ravi et al., 2018 [11]	Jung, 2017 [12]	Current study
Age and sex	8 years, male	22 years, male	23 years, male	35 years, male	58 years, female
Presenting manifestations	Fever, yellowish discoloration of skin and eye, and vomiting	Fever, yellowish discoloration of skin and eye, vomiting, and diarrhea	Fever, yellowish discoloration of skin and eye, loose stools, abdominal pain, and vomiting	Nausea, abdominal discomfort, and decreased urine output	Nausea, vomiting, and yellowish discoloration of skin and eyes
Hepatitis panel	HAV IgM positive	HAV IgM positive; HEV IgM, HBsAg, and anti-HCV negative	HAV IgM positive	HAV IgM positive	HAV IgM positive; HEV IgM, HBsAg, and anti-HCV negative
Total bilirubin (mg/dl)	51	40	40	10.29	7.9, increased to >20
ALT (U/L)	589	1853	2066	N/A	4792
AST (U/L)	341	2016	1753	N/A	5228
ALP (U/L)	N/A	189	N/A	N/A	313
Creatinine (mg/dl)	4.3	3.9, increased to 7	8.5	14.30	2.8, increased to >8
Renal biopsy finding	ATN with interstitial edema and inflammation	Interstitial edema, with tubules containing pigment casts	ATN with interstitial edema	Bile casts in renal tubules. Mononuclear cell infiltration and fibrosis in the interstitium	Mild interstitial edema. Injury to the tubular epithelium. Pigmented casts in the tubular lumen
Stains used	Fouchet stain was positive	Fouchet stain was positive	"Special stain" showed bile cast within the tubular lumen	N/A	Fouchet stain positive
Required hemodialysis (yes/no)	Yes	Yes	Yes	Yes	Yes
Follow-up duration	Three months	Ten weeks	N/A	N/A	Three months
Follow-up ALT (U/L)	146	120	175	N/A	17
Follow-up AST (U/L)	130	170	273	N/A	23
Follow-up creatinine (mg/dl)	0.2	2	2	N/A	0.8
Follow-up total bilirubin (mg/dL)	0.9	1.1	1.1	N/A	1.4

Kidney biopsy is considered the gold-standard method for diagnosing BCN [13]. A recent review by Tinti et al. [14] showed that several cases of CN were diagnosed via the identification of bile casts on renal biopsy, underscoring the necessity of using histological identification as the gold standard for diagnosing BCN. Interestingly, Chediak et al. [15] reported that transjugular renal biopsy (TJRB) is a better option than transcutaneous renal biopsy because the latter approach reaches the renal cortex while the former can identify lesions in the distal nephron where bile casts appear to be developing. The Hall (or Fouchet) histochemical stain shows green to yellow bile casts obstructing the renal tubules (Figure 2) [16]. All cases in our review (Table 2) underwent renal biopsy, and three specifically mentioned Fouchet stain. Tinti et al. [14] suggested that low albumin levels and acidosis may serve as bile cast-promoting factors (BCPFs). This study also suggested that bile casts in urine sediment analysis could aid diagnosis. In addition, it is essential to distinguish hepatorenal syndrome from BCN since both disorders occur in patients with liver disease. Nayak et al. [6] aimed to study the incidence of BCN at postmortem autopsies in patients admitted with a diagnosis of acute kidney injury secondary to hepatorenal syndrome. BCN was identified in 72.1% of patients with acute or chronic liver disease and 27.4% of patients with decompensated cirrhosis [6].

There are currently no established treatment guidelines for BCN. Treatment aims to lower bilirubin levels to prevent kidney dysfunction [4]. Hemodialysis is often used, especially when the patient needs it to improve kidney function [4]. All cases in the review (Table 2), including our patient, required hemodialysis with improvement in the clinical symptoms and laboratory values. In some instances, however, hemodialysis does not lower bilirubin levels sufficiently [4]. In these situations, other extracorporeal methods can be used. These include molecular adsorbents recycling system (MARS), coupled plasma filtration adsorption (CPFA), and plasmapheresis (XXII) [7]. Plasmapheresis has reportedly been used successfully in patients with BCN [7]. In addition, certain drugs, including steroids, cholestyramine, ursodeoxycholic acid, and lactulose, have been suggested, but these have been proven to be minimally effective [4]. However, ursodeoxycholic acid has been shown to alleviate CN in mice [2].

## Conclusions

BCN or CN is a relatively rare diagnosis resulting from direct toxic tubular cell injury, obstructive nephropathy, or hemodynamic compromise. We hope this case report encourages physicians to maintain a broad differential diagnosis in hyperbilirubinemia and acute kidney injury cases. Such patients should be evaluated for BCN, and renal biopsy is indicated. Therapy involves lowering bilirubin levels, but patients also often require hemodialysis.
